# Post-harvest evaluation of the effect of foliar and edaphic applications of silicon in pre-harvest of rose cv. ‘Brighton’

**DOI:** 10.1080/15592324.2025.2465234

**Published:** 2025-02-17

**Authors:** Eduard Machado López, Aquiles Darghan, Víctor Julio Flórez Roncancio

**Affiliations:** Facultad de Ciencias Agrarias, Universidad Nacional de Colombia, Bogota, Colombia

**Keywords:** Cut flowers, ethylene, flower longevity, GEE methodology, profile analysis

## Abstract

The longevity of the rose stem is often affected by the rate of respiration and the evolution in ethylene production, which also favors the development of *Botrytis*. Silicon is involved in plant defense, and its application could be a strategy to improve disease control. This research evaluated the effect of foliar and edaphic applications of silicon on the life of the Brighton rose using three sources of liquid silicon applied every 2 weeks in three foliar and edaphic conditions and one control. After harvest, the fresh mass loss, ethylene concentration, O_2_ consumption and CO_2_ evolution were measured. The number of fallen petals was counted, and the severity of the Botrytis infection was evaluated. The biomass loss of the floral stem was analyzed with profile analysis. For the evaluation of the change in values of O_2_, CO_2_ and ethylene, a multivariate semiparametric analysis of variance analysis was used and the generalized estimating equation methodology for the longitudinal binary response of severity. It was found that the soil treatment with lower potassium and soluble silicon was associated with a decrease in ethylene concentration as well as also turned out to be the one that best controlled *Botrytis* in post-harvest.

## Introduction

Roses (*Rosa* × *hybrida* L.) are the main exportable crop of all cut flowers worldwide.^1^ In Colombia, the production of roses generates about 200,000 formal jobs annually; 65% of the workers are rural women, especially in the Bogotá savanna and eastern Antioquia. A limiting factor of the production of fresh flowers is floral longevity in post-harvest. The senescence of flowers, usually related to the aging of petals, is an attribute of great interest and importance.^2^ Among the indicators that determine the longevity of cut flowers are the fall of petals and the change of color, as well as the dehydration of flowers and leaves, which depend on the sensitivity of the cultivar to ethylene.^3^

The vase life of roses is relatively short and dependent on variety, however it can be in the range of 8 to 15 days and is closely related to early water imbalance and the effect of ethylene.^4,5^ Flower stem wilting is caused by vascular blockage by embolism or microorganisms.^6^ At postharvest, other changes in floral stem physiology should also be considered, such as the increased production of reactive oxygen species (ROS) production and ethylene release, both of which impact floral senescence.^7^ Aminoethoxy-vinyl-glycine (AVG) inhibits the synthesis of 1-methylcyclopropene (1-MCP) in the action of ethylene; therefore, with the application of these compounds a longer life is achieved in cut roses of vases.^3^ Decreased water uptake, carbohydrate consumption, increased respiratory activity, and ethylene production are signs of floral stem senescence; and, as this maintains intense respiratory activity, they deplete the already limited carbohydrate reserves in the tissues.^8^

In perishable products, such as cut flowers, *Botrytis* is a relevant fungal pathogen that, by producing lesions on the petals, reduces their vase life and consequently the market value of the flowers. Infection with this necrotrophic pathogen is one of the most important reasons for consumer rejection of rose flower stems that might result in financial losses for the growers.^4^

The gene expression of ethylene biosynthesis maintains a linear relationship with the incidence and severity of *Botrytis cinerea*, positively affecting its development and dissemination. Likewise, during flower storage and transport, the hormone (ethylene) may also increase disease severity and, therefore, petal wilting.^9,10^

In terms of plant mineral nutrition, among the essential elements, the microelement silicon (Si) has recently gained prominence. Silicon is taken up by the plant in the form of monosilicic acid (H_4_SiO_4_), which polymerizes into silica gel or amorphous silica forms in the intercellular spaces of roots and in the cell walls of leaf and bract cells.^11^ The H_4_SiO_4_ enters the cell through the Lsi1 transporter, which belongs to the major intrinsic protein (NIP) family; and, it is exported, by efflux, to the aerial part of the plant by the Lsi2 silicon transporters. Some species maintain the Lsi6 transporter in leaf parenchyma cells, which helps move silicon through the vascular bundle.^12^ Silicon is considered a beneficial element for stimulating tolerance against biotic and abiotic stresses by neutralizing ROS and regulating different metabolic pathways, like the synthesis of jasmonic acid, enzymatic, and non-enzymatic antioxidants, and osmoprotectants.^13^

Silicon applications improve the defense mechanism of plants in agricultural crops, which can become a strategy to improve disease control.^14^ Wang *et al*.^15^ also found genetic evidence in the induction of salicylic acid and, thus, the defense mechanism that this hormone activates with high silicon content. Meanwhile, treatments with silica nanoparticles can improve longevity and postharvest quality of cut roses by reducing lipid peroxidation and stimulating antioxidant metabolism, thus, preserving membrane integrity.^16^

Recognizing that the vase life of cut roses is relatively short, typically five to eight days, it is evident that postharvest losses are a threat to profits in the floriculture industry.^17^ Therefore, preserving postharvest quality through a nutritional approach is of economic and environmental importance. With nutritional management that strengthens plant defenses against fungal diseases, a more environmentally friendly agronomic management is possible by reducing the number of fungicide or other pesticide applications. This research evaluated the effect of preharvest foliar and edaphic applications of silicon on the postharvest of roses cv. ‘Brighton’. In general, the application of silicon to the soil, especially soluble potassium in water 140 g L^−1^ and water-soluble silicon 322 g L^−1^ decreased ethylene production, generating a reduction in the severity of gray mold on the flower stems of cut roses cv. ‘Brigthon’.

## Materials and methods

### Plant material and location

The study was conducted on a farm dedicated to the production of cut flowers for export, located in the northwest of the savanna of Bogotá, the municipality of El Rosal, Cundinamarca, 4°50’45.402’‘N, 74°14’33.807’’ W between the road El Rosal – Subachoque, minimum and maximum temperatures of 8°C and 23°C and average PAR radiation of 280.6 μmolm^−2^ s^−1^, maximum relative humidity of 80% and a minimum of 68% average month. The trials were conducted in a millennium greenhouse with a metal structure and a conventional plastic cover without attachments, with a maximum height of 6.80 m and 4.30 m on the sides. The experiment was established in a seven-year-old commercial crop with plants of the Brighton variety grafted onto the Natal Briar rootstock, planted at a density of 7.2 plants m^−2^ in beds of 32 m × 0.9 m, considering 178 beds per hectare. The crop was established in soil characterized by sandy loam and a pH 6.62. The variety was selected for its high susceptibility to *Botrytis* fungus. On average, an irrigation volume of 675 L/bed/week was applied, and nutritional and phytosanitary management was carried out as routine in a rose crop.^18^

### Treatments

Three liquid silicon sources were used in the form of potassium silicate in nutrient solution: 1) 322 g L^−1^ of soluble silicon; 2) 360 g L^−1^ of total silicon; 3) 330 g L^−1^ of soluble silicon. Each silicon source was applied by a foliar spray (SF) and an edaphic form (SS) separately. Also, a control treatment (CT) was used where there was no silicon application. Seven treatments were established, and in six of them, silicon was applied in the form of potassium silicate (K_2_SiO_3_) from three different commercial sources, as described in Table 1. We applied the doses recommended by the commercial companies for the cultivation of roses. The names of the products are not given for confidentiality. Each treatment was conditioned in a greenhouse consisting of eight beds, and in greenhouse, there were four replicates, the experimental unit being composed of 10 plants chosen at random in the middle part of the beds, which had at least two 2-week-old flower stalks duly labeled. Two weeks after the pruning program, applications were started every 2 weeks, until five applications were reached, for subsequent post-harvest evaluation.

**Table 1.** Treatments used on rose plants cv. ‘Brighton’ subjected to silicon applications.

Treatment	Source	Composition	Method of Application	Dose (mL L^−1^)
CT	Control		conventional management^1^	N/A
SF1	Fertilizer 1	Soluble potassium in water 140 g L^−1^ and water-soluble silicon 322 g L^−1^	Foliar^2^	1
SS1			Edaphic^3^	0.08
SF2	Fertilizer 2	Water-soluble potassium 100 g L^−1^ and total silicon 360 g L^−1^	Foliar	1
SS2			Edaphic	0.08
SF3	Fertilizer 3	Water-soluble potassium 170 g L^−1^and water-soluble silicon 330 g L^−1^	Foliar	1
SS3			Edaphic	0.08

CT: standard fertigation formula of the farm without silicon: SF: standard fertigation formula of the farm and foliar spray of silicon (7 L per bed equivalent to 1,246 L/ha-1); SS: standard fertigation formula of the farm and soil application of silicon (135 L per bed equivalent to 24,030 L/ha-1).

### Travel simulation

Since this is a perishable export product, before determining the variables proposed for this trial, it was necessary to subject the plant material to a travel simulation, which consists of storage and transport periods that simulate the conditions to which the flower stalks are subjected during their itinerary to foreign destinations. For this activity, between 09:00 h and 11:00 h, we harvested six flower stalks per treatment in each of the four replicates, for a total of 168 flower stalks, which were hydrated in the field with an aqueous solution (pH = 7.0) based on sodium hypochlorite (3 mL L^−1^) for 20 min. We then transported the plant material in cardboard boxes to the postharvest laboratory of the Faculty of Agricultural Sciences of the National University of Colombia, Bogotá, where, in a controlled environment, we carried out a three-day storage and transport simulation at 3°C.

### Determination of fresh mass loss of flower stalks

Once the floral stems were arranged in the laboratory after the travel simulation, we selected by repetition three of the six stems, which were cut to the same length of 10 cm.^19^ We conditioned these stems in vases on laboratory benches with a sucrose and sodium hypochlorite-based solution, at a concentration of 45 mm and 200 mg L^−1^, respectively. The laboratory conditions were as follow: relative humidity between 60% and 70%, constant light between 4 and 50 μmol m^−2^s^−1^ (11-h natural light and 13-h artificial light), and an ambient temperature between 14°C and 16°C during the day and 8°C and 10°C at night, ventilated by opening doors and windows and the use of a fan for air recirculation. Using a digital balance, we determined the loss of fresh mass in grams for each set of three flower stalks and, consequently, the approximate water consumption by noting the difference between the initial mass and the final mass of each treatment.

### Measurement of ethylene evolution

We used the same flower stalks arranged in the hermetic chamber system from which a 5 cm^3^ gas sample was extracted with a syringe to determine the ethylene concentration (C_2_H_4_). For this purpose, we used an Agilent 7890 gas chromatograph (Agilent Technologies Inc., Santa Clara, CA, USA) equipped with a flame ionization detector with the use of helium as a carrier gas at a rate of 7 mL/min^−1^ and dry air and hydrogen as combustion gases at a rate of 300 and 40 mL/min^−1^. A standard ethylene nitrogen base of 101 µL L^−1^ (Linde Colombia S.A., Bogotá, Colombia) was used. For the use of the chromatograph, it was ensured that it was pure, that the inlet pressure was set, that it dried and that it was free from impurities. This gas was mixed with nitrogen (Linde Colombia S.A., Bogotá, Colombia) to obtain different concentrations in the range of 0–10 μL L^−1^ and the ethylene generation rate (r) was calculated using equation 1.^20^

(1) $$r=\frac{V}{W}\frac{\mathrm{\Delta y}}{\mathrm{\Delta t}}$$

where Δy represents the change in ethylene mole fraction during the times of interest (which can be that of two successive evaluations), thus $\frac{\Delta \;y}{\Delta t}$ represented the rate of ethylene production in the time interval *V* representing the free volume of the chamber with the flower stalk inside (cm^3^), and *W* representing the mass of the flower stalk (kg).

### Determination of O_2_ and CO_2_ evolution

To determine O_2_ consumption and CO_2_ evolution in a hermetic system, we used the MOCON Checkpoint 3EC device and measured the percentages of both gases using infrared sensors. Previously, flower stalks were confined for 2 h in transparent airtight chambers (Felli®, Taipei, Taiwan), with a hermetic sealing system and a modified lid for internal gas sampling through a volume headspace of 2,210 cm^3^. The needle of the MOCON device was inserted through a rubber seal at the top of the airtight chamber to collect the sample. During the evaluation, we made six data collections every other day in each of the treatment replicates. We calculated the O_2_ and CO_2_ rates associated with respiration using the same equation 1, only now Δ y substituted for the mole fractions of O_2_ and CO_2_ determined at the preferred time interval.

### Petal fall

For the determination of vase life, the variable petal fall was also evaluated. We counted every 2 days during 14 days the number of petals that fell from the flower head. We obtained the median of this variable by accumulating the information from three flower stems in each of the treatment replicates.

### Determination of the percentage of disease severity of botrytis

To determine the degree of severity of *Botrytis* during postharvest, we used the severity scale proposed by Medina *et al*.^21^; evaluating the degrees of severity as 0, 1, 2, 3 or 4, we assigned the percentages of petal area affected from 0% to 1% to 25%, 26% to 50%, 51% to 75%, and 76% to 100%. We took measurements every other day during the floral longevity evaluation.

### Experimental design and data analysis

Our study involved several experiments that implied the use of different statistical techniques either due to the univariate or multivariate nature of the response or by the type of variable (discrete or continuous). The entire study consisted of seven treatments, four replicates of the experimental unit, and three observation units per experimental unit. To evaluate the loss of floral stem biomass, the research design used repeated measures with a between-subjects factor formed by the seven treatments described above and a within-subjects factor associated with the evaluation times in the different phases (2, 4, 6, 8, 10 and 12 days). As the study is longitudinal, the fresh mass loss profiles in the floral stems were first plotted, and the profile analysis was used to evaluate the parallelism, coincidence or horizontality of the profiles.^22^ We analyzed this data with profile analysis (profileR library).^23^ In the presence of parallelism (no coincidence), the pattern of the profiles is monotonic for each treatment; to extract more information about the nature of the parallelism, the area associated with progress to loss was calculated in a stepwise fashion for each repetition to eliminate the effect of time and just compare treatments.^24^ Therefore, the analysis was simply an analysis of variance for a single factor using Tukey’s test (HSD) to make a posteriori comparisons of variance analysis, available in the stats and agricolae libraries.^25,26^

In the experiment associated with the measurement of $O_{2}\;$and $CO_{2}$ and ethylene, we used the multivariate semiparametric approach of variance analysis. The results generated an analysis of variance table that involved the simultaneous analysis of the three responses to evaluate the main and interaction effects of the factors. This analysis was implemented in the MANOVA.RM library of RStudio version 4.2.2.^27^

For the experiment associated with the count of fully detached petals, we implemented the statistical analysis from a descriptive point of view using a line plot discriminated by treatment and evaluation time. Finally, in the experiment associated with the measurement of *Botrytis* infection, we used the generalized equations estimation (GEE) method under the model of absence of interaction between treatments and time (elimination by null effect), with a table of probabilities estimated by treatment and time. The methodology allows to evaluate repeated measures in binary response and estimate the likelihood of infection by treatment over time (geepack library).^28^

## Results

**Figure 1.**
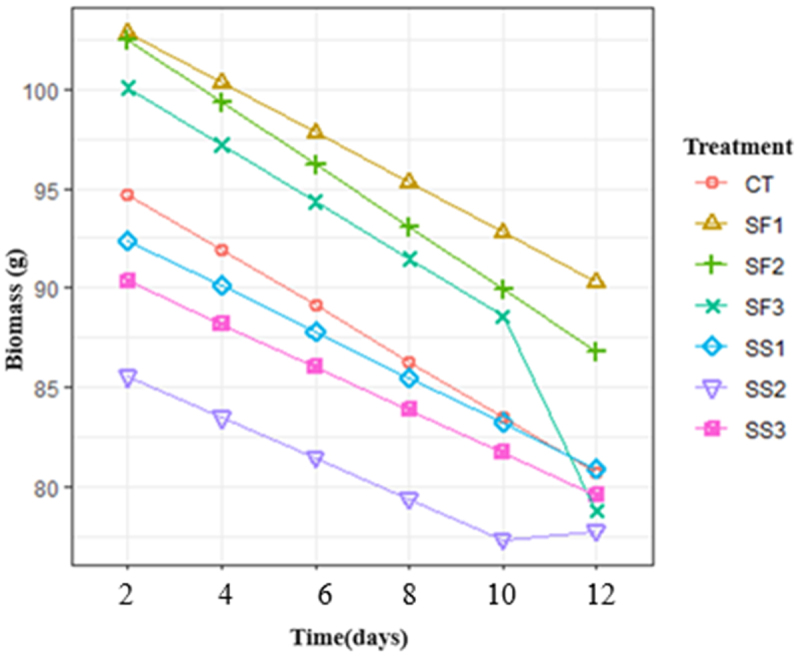
Profiles of fresh loss of flower stem mass of rose cv ‘Brighton’, in plants subjected to foliar and edaphic silicon applications.

Initially, the loss of fresh mass in flower stalks was estimated to quantify dehydration from treatments. The results showed 19.5% for the control and 14.0%, 14.2%, 13.7% and 13.4% for the SF1, SS1, SS2 and SS3 treatments. The data provided evidence in favor of the hypothesis of parallelism in the profile analysis (Figure 1) so we calculated the areas under step curve from which the analysis of variance for this measure associated with the progress of fresh mass loss and obtained a posteriori comparison of area means using Tukey’s method (Table 2). Figure 1 shows the change of the variable over time and the parallelism in such profiles, with some variations without relevance in the SF3 and SS2 treatments, especially in the last measurement. Thus, we inferred a statistical difference between some of the treatments, where especially treatments SS2, SS3 and SS1 obtained the lowest percentage of mass loss; however, in the determination of the area under the curve for each treatment, the homogeneous groups in Table 2 were generated, with the most contrasting treatments being SS2 and SF1. The rest of the groups shares equality with SS2 or SF1.

**Table 2.** Average area under the curve in the measurement of fresh mass loss in flower stems of the rose cv ‘Brighton’ from plants subjected to foliar and edaphic silicon applications.

Treatment	SS2	SS3	SS1	CT	SF3	SF2	SF1
Average	160.83	169.91	173.28	175.42	185.79	188.82	193.12
Group	a	ab	ab	ab	ab	ab	b

CT: Control treatment, SF1: Silicon 1 foliar, SF2: Silicon 2 foliar, SF3: Silicon 3 foliar, SS1: Silicon 1 edaphic, SS2: Silicon 2 edaphic, SS3: Silicon 3 edaphic. The letters represent the homogeneous groups resulting from the comparison of the areas under the profiles using the Tukey method.

**Figure 2.**
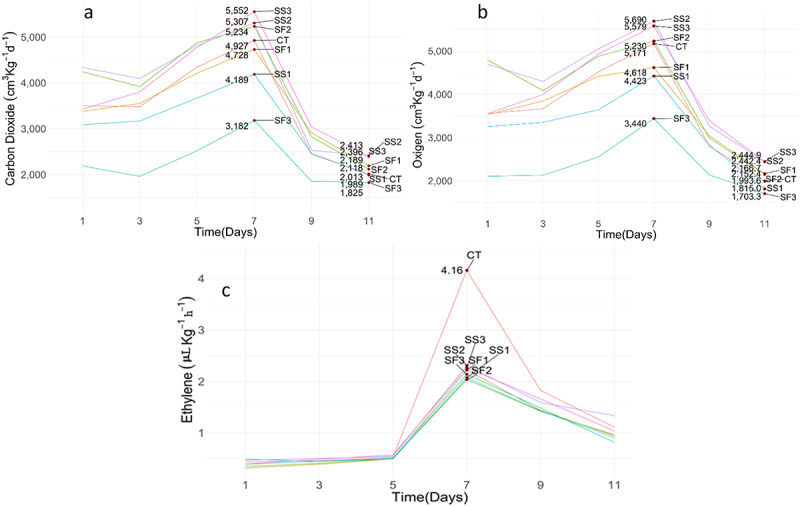
(a) carbon dioxide (CO_2_) production rate, (b) oxygen (O_2_) consumption rate, and (c) ethylene (C_2_H_4_) generation rate in flower stems of rose. Treatments: CT = control treatment, SF1 = foliar silicon 1, SF2 = foliar silicon 2, SF3 = foliar silicon 3, SS1 = silicon 1 to soil, SS2 = silicon 2 to soil, SS3 = silicon 3 to soil.

Table 3 shows the statistical analysis for variables C_2_H_4_, O_2_ and CO_2_ (multivariate). The absence of interaction allowed us to consider the rejection of the null hypothesis of zero effect of time and close differences between the responses of the treatments (*p* = 0.095). Figure 2 shows maximum values after 7 days of evaluation for all treatments. The highest point in ethylene generation was achieved in the seventh day (4.16 cm^3^Kg^−1^h^−1^), with all treatments below control. On the last day of assessment (day 11), it was observed that only SS2 treatment was above control as regards ethylene production, with about 20% more, while SF3 and SS1 were below control with 26% and 18%.

**Table 3.** Table of the modified multivariate semi-parametric analysis of variance in repeated measures with bootstrap p-value estimation.

Factor	Statistic	Param-Between Subjects (Multivariate)
Treatment(T)	168.81	0.095
Time(t)	981.63	<0.001
Interaction(T×t)	55.88	0.442

**Figure 3.**
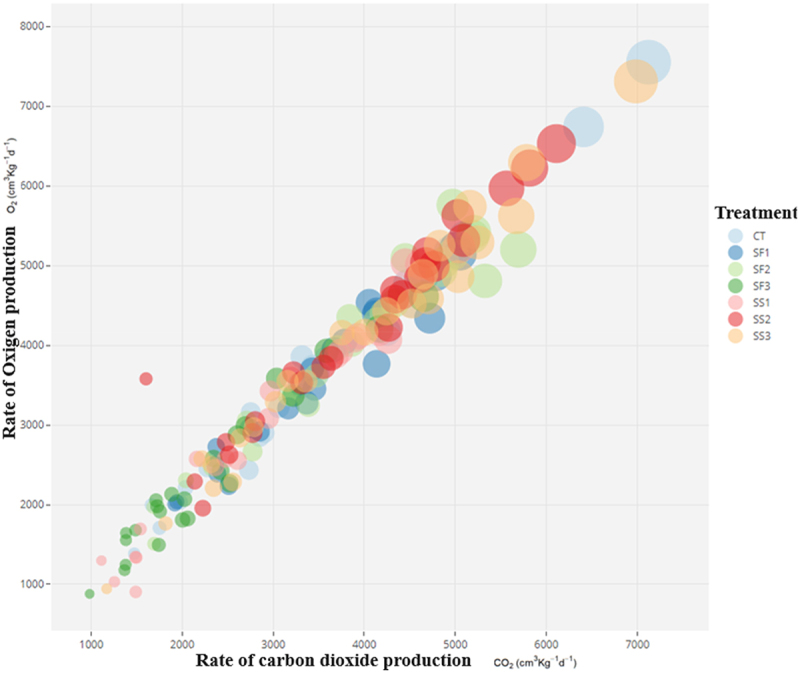
Comparison of ethylene (C_2_H_4_) (the bubble size represents their values), oxygen (O_2_) and carbon dioxide (CO_2_) production rates in flower stems of the rose cv ‘Brighton’, in plants subjected to foliar and edaphic silicon applications.

Although complementing with the descriptive result in Figure 3, as the p-value = 0.095 was not extreme, the highest coincidence is clear in ethylene (with a separation from the control treatment in the seventh evaluation), but it was not so evident in O_2_ and CO_2_, where SF3 and SS1 showed the lowest production rates, while the rest of the treatments were higher and quite similar.

For CO_2_ generation, Figure 2a showed in the first measurement (day 1) an increase with respect to the control of 24% and 21% in the first measurement for the treatments SS2 and SF2. Treatments SF3 and SS1 showed a below of 37% and 12%, respectively, compared to the control.

Furthermore, at the highest point (day 7) treatments SS3 with 13% and SS2 with 8% were above the control, while treatments SF3 and SS1 were below in CO_2_ generation with respect to the control by 35% and 15%, respectively. On the last day of evaluation, day 11, the CO_2_ generation of the treatments that were the highest was SS2 with 20% and SS3 with 19% above the control, while SF3 and SS1 were below the control with 9% and 1% respectively.

When relating the three responses in a bubble diagram (Figure 3), the linear relationship between O_2_ and CO_2_ production is clear (with Pearson correlation of 0.98). It is also evident that the lowest ethylene concentrations occur at the lowest O_2_ and CO_2_ values, with maximum ethylene values in the control and SS2 and minimums in SF1, SF2 and SS1.

The rate of ethylene production had an increasing trend in all treatments until day 7 and then had a drop with variable rates until the last evaluation date. As explained above, the data showed evidence in favor of the null hypothesis of null effect of the three responses attributable to the treatments.

From a descriptive point of view, Figure 2b showed that in the evaluation for day 1, the SF2 and SS2 treatments had more O_2_ consumption than the control by 35% and 32%, while the SF3 and SS1 treatments were below the O_2_ consumption of the control by 41% and 9%. From day 1, oxygen consumption increased until day 7, when it reached its maximum peak; on day 7, treatments SS2 and SS3 obtained the highest consumption with 10% and 8%, compared to the control; the lowest consumption was in treatments SF3 with 33%, SS1 with 15% and SF1 with 11% compared to the control. In the last evaluation, on day 11, the highest oxygen consumptions were in treatments SS3 and SS2 with 23% each above the control, while SF3 and SS1 were below the control with 15% and 9%.

**Figure 4.**
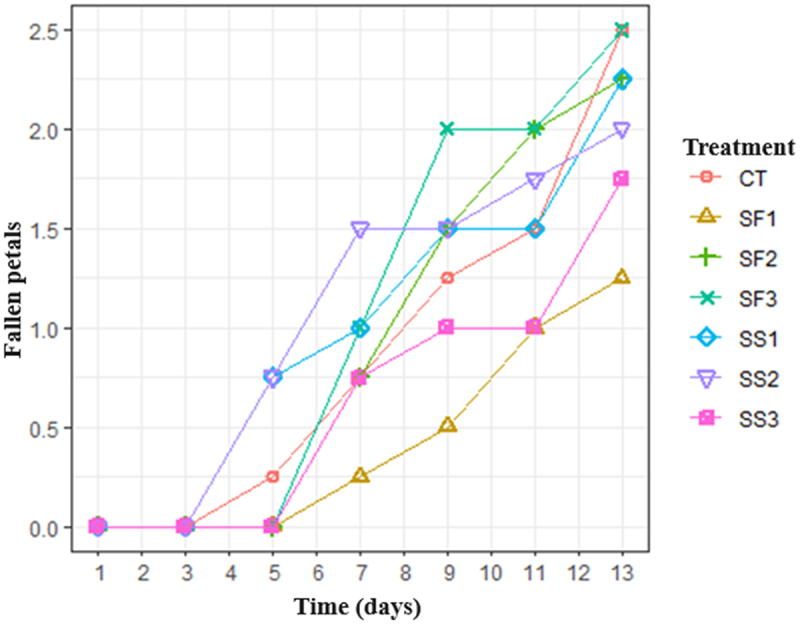
Average number of fallen petals on three flower stalks of rose cv. ‘Brigthon’ during postharvest evaluation. Petal drop started after 13 days of evaluation.

Petal fall is one of the most important factors in the vase life of roses. The foliar silicon treatment 1 (SF1) was the best performance among all treatments with 1.25 petals dropped during the 13 days of evaluation, followed by soil applied silicon 3 and 2 (SS3 and SS2) with 1.75 and 2 petals dropped, respectively. The control treatment and foliar silicon (2) showed more petals dropped with 2.5 and 2.25, during the evaluation (Figure 4).

**Figure 5.**
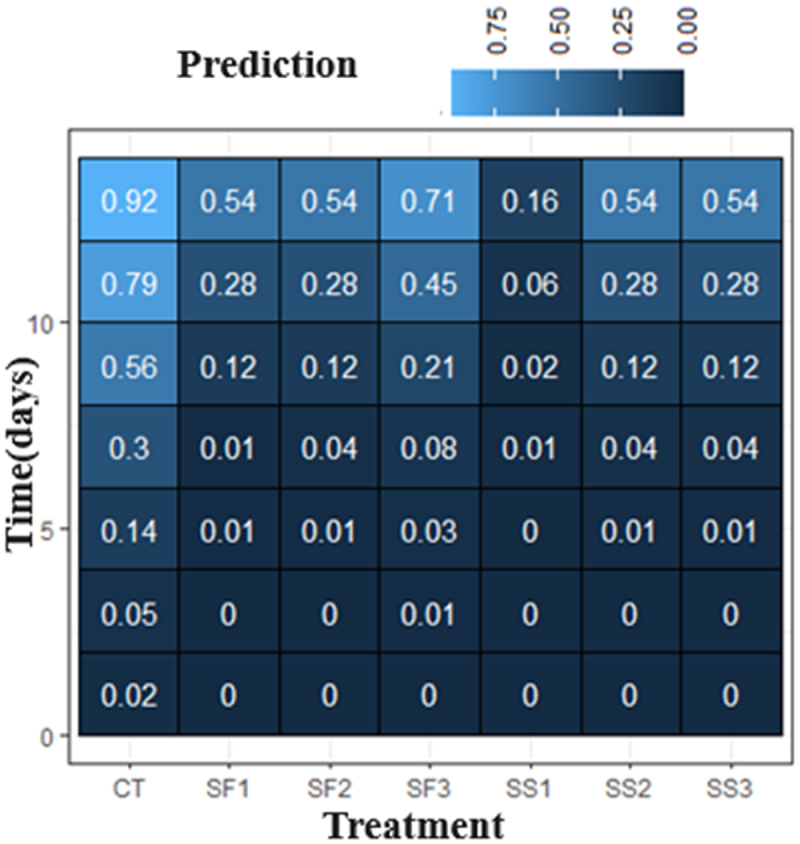
Distribution of prevalence by treatment and time of evaluation (days).

Figure 5 showed the distribution of the probability of incidence of gray mold, caused by *Botrytis cinerea*, where the darker the color of the cell, the lower the probability of being affected by the disease in terms of the lesion on the flower petal. Accordingly, the detection of the appearance of *Botrytis* was unlikely on days 1, 3 and 5 of evolution. Meanwhile, on day 7, the identification of visual symptoms of the infection began, especially for the control treatment and in SF2. On day 9, a higher probability (56%) in the control and 21% in SF3 was detected; on day 11, there was a highly probable (79%) that petal lesions of the disease under study could appear in the control treatment and there was a moderate probability in SF1 and SS2, unlikely in SF2 and SS3, and very unlikely in SS1, with only 6%. On the last day of evaluation, it is highly probable that the control had *Botrytis* damage, moderately probable with SF1, SF2, SF3, SS2 and SS3, and unlikely in SS1.

The model finally selected, which allowed estimation of the probabilities of severity, yielded the following statistical meanings for each effect (Table 4).

**Table 4.** Distribution of *p* values for levels associated with treatments and time effect. The intercept represents the excluded level (control).

Coefficient	Estimate	Std.Err.	Wald	p-value	Mean response
Intercept	−4.69	0.81	33.26	8.1e-9	0.39
Time	0.55	0.08	44.53	2.5e-11	0.17
SF1	−2.27	0.81	7.88	5.0e-3	0.14
SF2	−2.27	0.99	5.21	2.3e-2	0.14
SF3	−1.53	0.82	3.51	0.06	0.21
SS1	−4.09	1.14	12.76	3.5e-4	0.04
SS2	−2.27	0.81	7.88	5.0e-3	0.14
SS3	−2.27	0.86	6.93	8.5e-3	0.14

The mean severity response was estimated from the model. The time value was obtained from the average of all factor levels associated with treatments.

## Discussion

The collection and storage of fresh cut flowers implies their deterioration, being a perishable product where the metabolism remains active generating biomass loss.^29^ The results obtained show a parallel of profiles and under their areas there was a decrease in fresh biomass between treatments due to the decrease in the area under the curve, with the lowest loss being group SS2 (water-soluble potassium 100 g L^−1^ and total silicon 360 g L^−1^) and the greater loss group is SF1 (Soluble potassium in water 140 g L^−1^ and water-soluble silicon 322 g L^−1^). These results are consistent with those reported by Jamali and Rahemi,^30^ who reported less loss of fresh mass in carnation flower stems treated with silicon at doses of 100, 150 and 300 mg L^−1^. Also, results of cv roses. ‘Vega’ and ‘Gold Star’ agree that silicon in foliar or soil application reduces the loss of fresh mass and increases the dry mass of roses after cutting.^16,29^

After harvest, flower stalks are exposed to a certain level of stress. The rose cv. ‘Vendela’ is a high producer of ethylene and generates peak emissions that may depend mainly on the level of stress to which the flowers are exposed.^19^ The results are similar to those obtained for the cv roses. ‘Brigthon’, where the foliar application of the three silicon sources used and with one applied to the soil (SS1) showed changes in time and very close differences from the statistical point of view despite obtaining a value *p* = 0.095 for the three responses associated with oxygen, ethylene and carbon dioxide, with maximum on the seventh day and with smaller differences between treatments for ethylene and more marked differences in O_2_ and CO_2_, with smaller values in SS1 (water-soluble potassium 140 g L^−1^ and water-soluble silicon 322 g L^−1^).

These results show that the application of silicon in pre-harvest is associated with the reduction of ethylene during the life of the pot of cut roses cv. ‘Brigthon’, which coincides with those reported by Jamali and Rahemi,^30^ who used a nutritive solution of silicon in the form of potassium silicate in the post-harvest of carnations, in doses of 100, 150 and 300 mg L^−1^, finding a significant reduction in ethylene production compared to the control, which associated with greater floral longevity.

Regarding the evolution of O_2_ and CO_2_, they showed the close differences from a statistical point of view attributable to treatments, especially those in which silicon is applied. Although the literature does not seem to show a clear agreement on the fact that the application of silicon affects respiration.^31^ Being strict in the obtained probability values we can show very surely even results, however, a *p* value as obtained in Table 3, especially when the analysis is multivariate, is to be cautious in simply concluding on the basis of this measure and omitting the profiles of Figures 2 and 3.

It is well known that ethylene reduction is one of the factors in the maintenance of floral quality because petal fall is delayed during vase life^19^ in cut rose cv. ‘Vendela’, which is susceptible to ethylene action, with petal abscission as the main effect. In our investigation, the application of silicon, regardless of the form of application and the concentration used, decreased the climacteric peak of ethylene, compared to the control treatment. Although there is evidence that silicon can indirectly inhibit ethylene synthesis under biotic stress conditions,^32^ there is still no clarity of this mechanism in decreasing ethylene production in senescence. Since the senescence of cut flowers can be slowed by increased production of reactive oxygen species detoxifying molecules^33^ and, one of the functions of silicon is to promote the synthesis of antioxidant enzymes,^34^ it is likely that senescence of rose var. ‘Brigthon’ was decreased with silicon application because of its impact on ethylene production. With foliar application of silicon in carnations, Jamali and Rahemi^30^ also obtained lower ethylene production in postharvest of this species.

Although the variable was counted with only a descriptive statistical analysis, the results show that only one of the treatments with silicon remained similar to the control treatment, while the other treatments showed lower numbers, with the best result for treatment with silicon from applied leaf source 2 and 1 (SF2, SF1), followed by edaphic application of source 1 (SS1). The fall of petals is related to the production of ethylene, as an indicator of floral senescence.^19^ Geerdink et al.^29^ also reported that, in the rose cv. ‘Vega’, the silicon improves the water condition of the petals by increasing turgor and decreasing water loss over time. Therefore, the silicon, by decreasing the production of ethylene, would delay the fall of the petals in the flowers of the rose var. ‘Brigthon’.

In banana, a species known as a silicon accumulator, silicon decreases ethylene production.^35^ Nikagolla et al.^36^ suggest that silicon treatment can be used as an alternative to the use of fungicides in the control of post-harvest fungal rot. For the authors, such treatment generates additional benefits, such as increased fruit firmness and delayed ripening with statistically significant decreases in CO_2_ and ethylene production.

The decrease in ethylene delays the symptoms of gray mold in roses. Ha *et al*.,^10^ using a treatment of 1-MCP, manage to delay the appearance of *Botrytis* by up to 8 days after inoculation, confirming the direct relationship between the evolution of the hormone and damage to the petals by the disease. In addition, silicon in the rose cv. ‘Smart’ would increase the expression of the *GLS5* gene, involved in the deposition of callose in the cell wall, which, together with the accumulation of silicon in the apoplast, generates a barrier that limits the expansion of powdery mildew *(Podosphaera pannosa)*, which could also limit *Botrytis* infection. In this experiment, all silicon treatments reduced the severity levels of gray mold and the probability of its appearance during the evaluation period; among them, treatment SS1 showed healthier petals and a lower probability of infection.

These results with soil-applied silicon could be related to its absorption and translocation in the root, which in several species occurs through the membrane transporters Lsi1 and Lsi2.^12^ Although in the literature there is still no evidence of such transporters in rose plants, the trend observed in the results of our research could be related to the activity of the transporters, on the grounds that a better effect of silicon was found when applied to the soil compared to foliar application. This agrees with Geerdink et al.^29^ Also, silicon can stimulate plant responses such as gene activation against adverse biotic and abiotic factors that cause oxidative stress in cells.^13^

## Supplementary Material

Supplemental Material
